# Perianal Abscess Caused by Urethral Foreign Body: A Case Report

**DOI:** 10.1002/iju5.70017

**Published:** 2025-03-13

**Authors:** Sayaka Hoshino, Kenji Obara, Kazutoshi Yamana, Fumio Ishizaki, Masaki Murata, Tatsuhiko Hoshii, Yoshihiko Tomita

**Affiliations:** ^1^ Division of Urology, Graduate School of Medical and Dental Sciences Niigata University Niigata Japan

**Keywords:** intellectual disability, perianal abscess, urethral foreign body

## Abstract

**Introduction:**

We report a case of perianal abscess that developed after the presence of a urethral foreign body for 15 months.

**Case Presentation:**

A 14‐year‐old boy with intellectual disability due to agenesis of the corpus callosum presented to our hospital with complaints of perianal erythema, swelling, and pain. The patient admitted to self‐inserting an allene fiber broom ear 15 months ago. A few days after the insertion, he was treated with antibiotics for hematuria and micturition pain. The symptoms resolved spontaneously 2 months later, although the foreign body was not removed. Computed tomography and magnetic resonance imaging demonstrated a perineal linear structure running anterior to posterior. The urethral foreign body was removed by cystourethroscopy and grasper.

**Conclusion:**

Medical professionals should be aware of the possibility of underlying cognitive and behavioral problems and should be vigilant in screening and treating individuals with refractory lower urinary tract inflammation or hematuria.

**Summary**

- In patients who have intellectual disability due to agenesis of the corpus callosum, medical professionals should be aware of the possibility of underlying cognitive and behavioral problems and should be vigilant in screening and treating individuals with refractory lower urinary tract inflammation or hematuria.

## 1 Introduction

The insertion of a foreign body into the urethra is more common in men than in women, and it can occur at any age [1]. The most common reason for urethral foreign body insertion is sexual gratification, but other reasons include psychiatric disorders [2]. We report a case of a perianal abscess that developed after the presence of a urethral foreign body for 15 months.

## 2 Case Report

**FIGURE 1:**
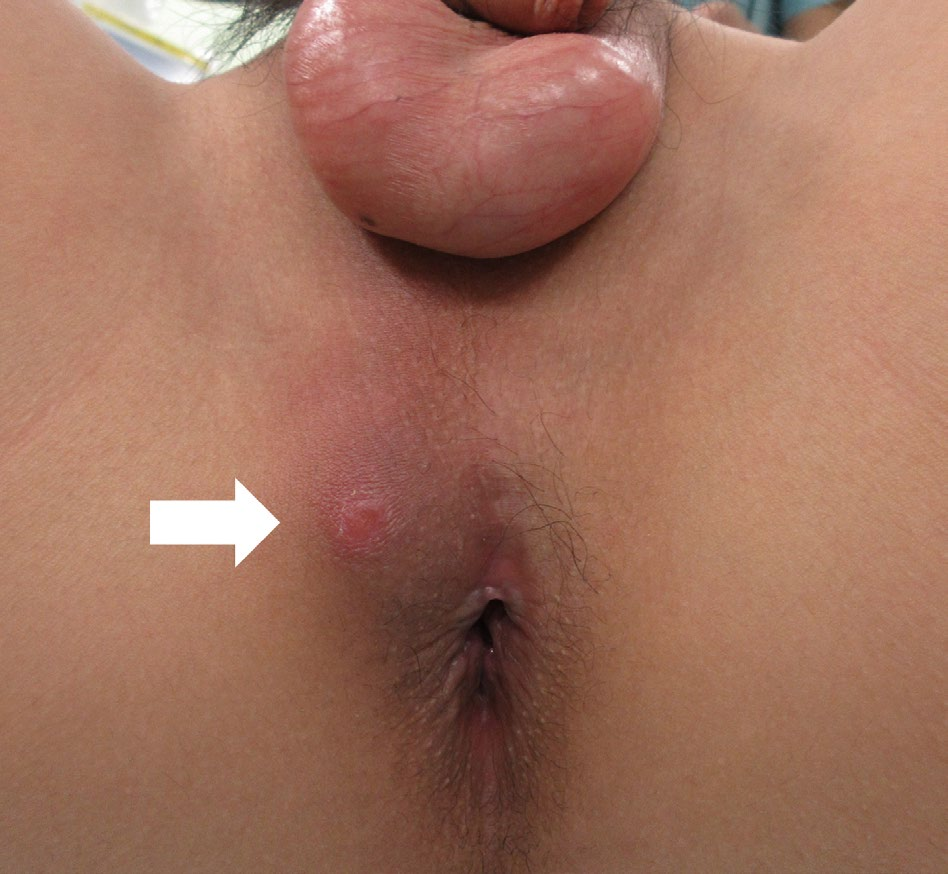
Perianal abscess with erythema, swelling, and pain (arrow).

**FIGURE 2:**
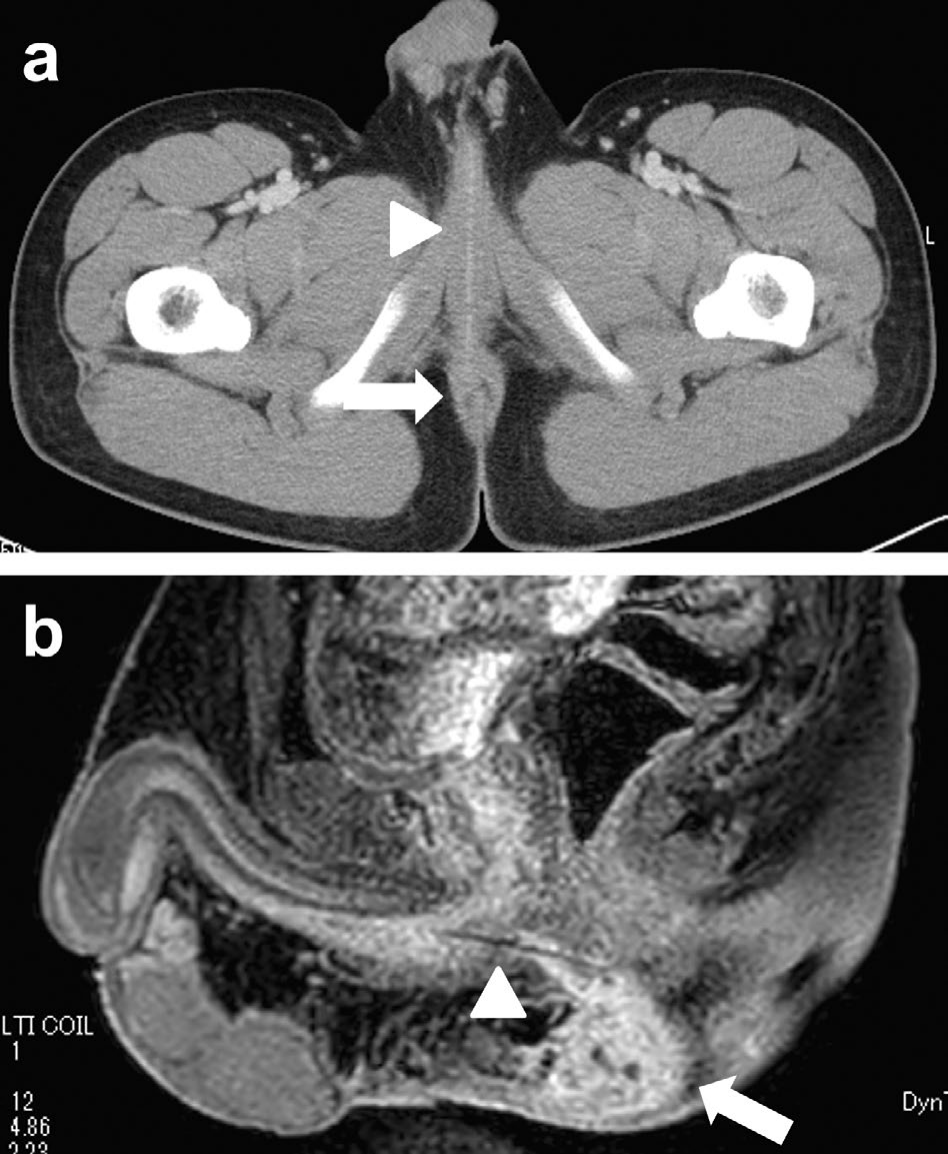
Computed tomography reveals the presence of a perianal abscess (arrow) and a perineal linear high‐density structure running anterior to posterior (arrow head) (a). Magnetic resonance imaging demonstrates a linear low‐intensity structure (arrow head), extending from the bulbar urethra to the perianal abscess (arrow) (b).

**FIGURE 3:**
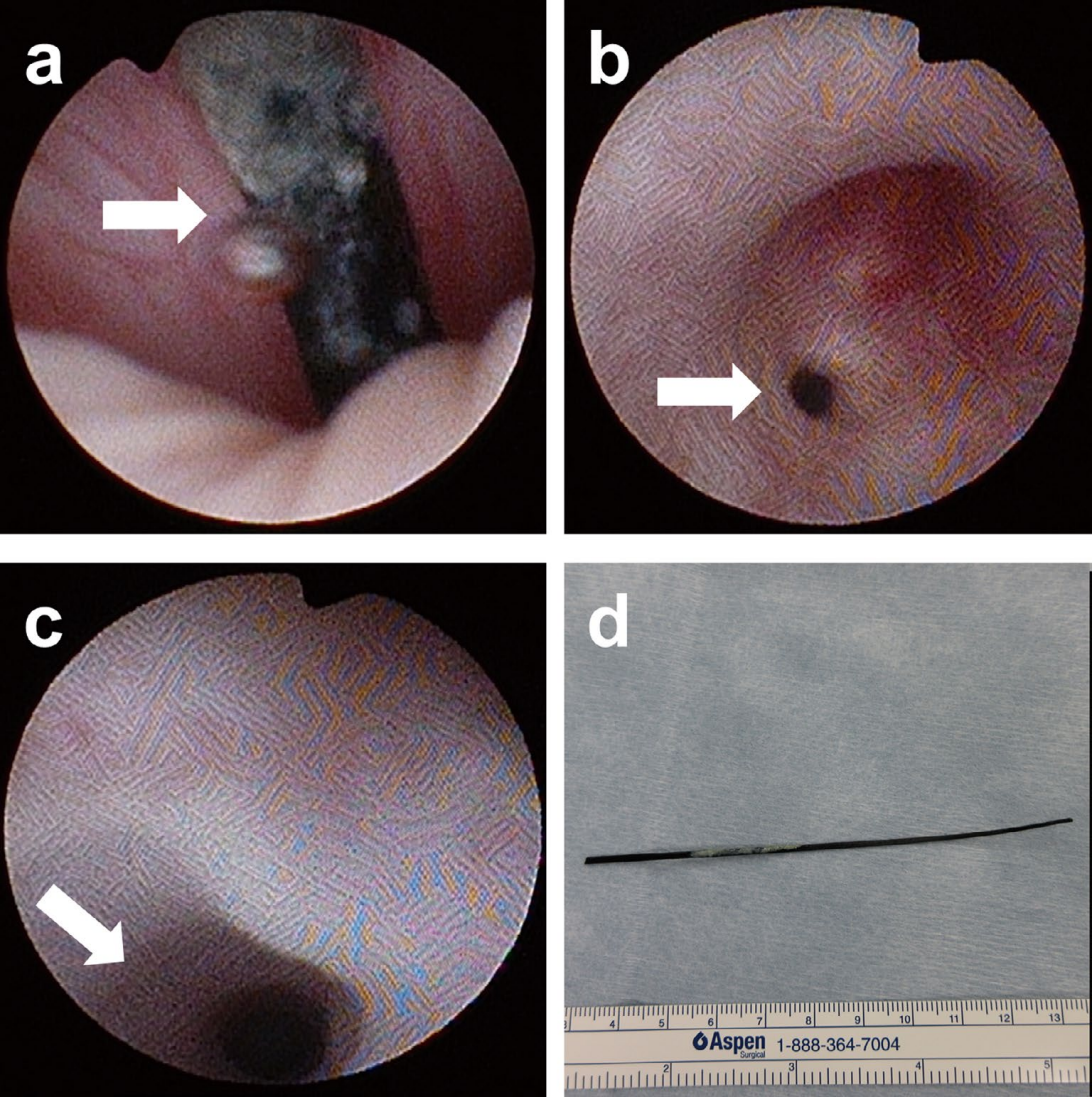
Urethroscopy showing a black and thin stick at the bulbar urethra (arrow) (a). The tract between the urethra and the abscess was observed to have epithelized (arrow) (b, c). An allene fiber broom ear measuring 10 cm in length (d).

A 14‐year‐old boy with intellectual disability due to agenesis of the corpus callosum presented to our hospital with complaints of perianal erythema, swelling, and pain (Figure 1). After a careful history taking, the patient admitted to self‐inserting an allene fiber broom ear 15 months ago. Allen fiber is a thin, strong fiber, and is often used as a broom material for this reason. A few days after the insertion, he was treated with antibiotics for hematuria and micturition pain. The symptoms resolved spontaneously 2 months later, although the foreign body was not removed. Computed tomography (CT) scan revealed a perianal abscess and a perineal linear high‐density structure running anterior to posterior (Figure 2a). Magnetic resonance imaging (MRI) also showed a linear low‐intensity structure from the bulbar urethra to the perianal abscess (Figure 2b). A colonoscopy was performed prior to surgery, which excluded the possibility of rectal injury. The foreign body was removed using a 10.8‐Fr cystourethroscope and grasper under general anesthesia (Figure 3a–d). After removal of the foreign body, pus discharge was observed during cystoscopy due to compression of the perianal abscess. The tract between the urethra and the abscess was observed to have epithelized. A Foley catheter was left in situ for 5 days. The culture result of the abscess was *Escherichia coli* . Antibiotics were administered until the abscess cavity disappeared.

## 3 Discussion

A perianal abscess is defined as a localized collection of pus in the tissues surrounding the anus. It is a common finding for there to be an abscess and a fistula‐in‐ano [3]. The occurrence of perianal abscess caused by the presence of a foreign body is relatively rare, especially urethral foreign bodies. Adolescents who insert foreign bodies into the urethra may present with a variety of symptoms, including pain, discomfort, urinary difficulty, urinary tract infections, and bleeding [4, 5]. These symptoms may prompt the adolescent to seek medical attention. However, many cases remain unreported due to feelings of embarrassment or shame. A diagnosis of a perianal abscess caused by a urethral foreign body typically necessitates a physical examination and imaging studies, such as a CT scan or MRI, to identify the foreign body and evaluate the extent of the abscess [1]. The standard treatment for a perianal abscess caused by a urethral foreign body is to remove the foreign body, drain the abscess, and administer antibiotics to treat any associated infection. In cases where a foreign body is not readily removable, surgical intervention may be required to facilitate its removal [2].

The corpus callosum is a band of nerve fibers that connects the two hemispheres of the brain, facilitating communication between them. When there is agenesis or damage to the corpus callosum, it can result in a range of neurological and cognitive deficits. One of the potential cognitive deficits is intellectual disability, which is characterized by significant limitations in intellectual functioning and adaptive behavior [6]. In this case, the patient presented with symptoms of hematuria and micturition pain a few days after the insertion of a foreign body into the urethra. The patient and his parents consulted several medical professionals. In most cases, the insertion of a foreign body into the urethra would have been recognized at this stage, and appropriate treatment would have been initiated. However, this information was not communicated to the physicians, and treatment with antibiotics and analgesics continued for approximately 2 months. It is important to note that there is no direct relationship between agenesis of the corpus callosum and a urethral foreign body; medical professionals should be aware of the possibility of underlying cognitive and behavioral problems and should be vigilant in screening and treating individuals with refractory lower urinary tract inflammation or hematuria in such patients.

When it was determined that a perianal abscess was caused by a foreign body in the urethra, the possibility of the foreign body having been inserted by someone other than the patient himself was a concern, and it was suspected that even if this was the case, the patient might not be able to give an accurate explanation. Careful interviewing is essential in cases of sexual abuse, especially involving people with intellectual disabilities, but such investigations are often challenging [7]. However, in this particular instance, the patient's family members provided that the patient had been collecting similar objects around the time the foreign body was inserted. Furthermore, there were no interpersonal problems at school or elsewhere, which supports the likelihood that he was the one who inserted the object, as the patient himself stated. Despite careful questioning, he did not provide a reason for inserting the allene fiber broom ear into his urethra. His parents were also unaware of the reason. Consequently, the question of whether the patient experienced sexual gratification remains unanswered.

## 4 Conclusion

We present a rare case of perianal abscess that occurred in a patient with agenesis of the corpus callosum, which developed after the presence of a urethral foreign body for 15 months.

## Ethics Statement

This study has been approved by a suitably constituted Ethics Committee of the institution. The registration number is 2015‐2641.

## Consent

Written informed consent for publication was obtained from the patient' guardians.

## Conflicts of Interest

The authors declare no conflicts of interest.
